# Stocking density, restricted trough space, and implications for sheep behaviour and biological functioning

**DOI:** 10.3389/fvets.2022.965635

**Published:** 2022-09-28

**Authors:** Bonnie T. Mayes, L. Amy Tait, Frances C. Cowley, John M. Morton, Brendan P. Doyle, Muhammad A. Arslan, Peta S. Taylor

**Affiliations:** ^1^School of Environmental and Rural Science, University of New England, Armidale, NSW, Australia; ^2^Jemora Pty Ltd., East Geelong, VIC, Australia

**Keywords:** live export, faecal glucocorticoid metabolites, lying positions, allometry, ruminant welfare

## Abstract

Stocking density and trough space allowance can potentially impact sheep welfare during live export voyages. The aim of this study was to assess the welfare implications for sheep housed at five allometric stocking densities, with either unrestricted or restricted trough space allowance. Merino wethers (*n* = 720) were housed in 40 pens of 18 heads for 18 days. Two 5-min continuous focal animal observations (n = 3/pen) were conducted on days 3, 5, 11, and 17. Scan sampling of standing and lying behaviours were conducted on the same days at hourly intervals. Live weights and immune cell counts were quantified at the start and end of the experiment, as well as faecal glucocorticoid metabolites (FGCMs), which were also assessed on days 6 and 12. Focal animals housed at higher stocking densities spent less time lying during one of the continuous observation periods, but no important effects on the overall number of animals lying or on the synchronicity of lying were evident. The scan sampling results indicated that the expression of some preferred lying positions was impaired at high stocking densities, and that high stocking densities also resulted in increased agonistic social interactions and displacement events at the start of the trial. There was a slight reduction in day 18 live weights for animals housed at higher stocking densities, but FGCM concentrations and immune cell counts were essentially unaffected. Trough space had no important effects on day 18 live weight, FGCM concentrations, or immune cell counts, and had limited effects on sheep behaviour. The lack of important impacts on biological fitness traits suggests that the behavioural responses observed were sufficient in allowing sheep to cope with their environment. However, we provide evidence that the provision of additional space is beneficial in reducing the time it takes for animals to adapt to their environment and to facilitate the expression of some preferred lying positions. While designed to emulate certain conditions relevant during live export voyages, some factors that may induce stress during this mode of transport were not present such as heat and ocean swell, so the conclusions must be interpreted in the context of the experimental conditions.

## Introduction

Australia's live sheep export trade is the world's largest live animal trade by sea (1). As community interest in animal welfare grows (2), there has been extensive public concern in recent years regarding the welfare of animals on live export ships (3). Arguably, space allowance is one of the most important determinants of animal welfare during transportation (4); it impacts an animal's ability to rest and be comfortable, its ability to thermally regulate, and ability to move around freely and perform certain behaviours as it desires (3).

Stocking densities during Australian live export voyages are, since 2018 (5), being determined using allometric principles, which describe relationships among physical measurements of an object and changes in the size or volume of the object, so these measurements can be used to describe spatial needs over a range of live weights (6). The allometric equation that states area = *k*W^2/3^ incorporates the live weight of animals (W) to estimate spatial requirements (4), where *k* represents a space allowance coefficient constant, and area is calculated in square metres. A range of *k-*values has been suggested by stakeholders to meet the welfare requirements of sheep in the live export industry. A *k-*value of 0.027 has been suggested to be sufficient for simultaneous lying but inadequate for animals to freely access resources (5). Indeed, some authors (4) state that a *k*-value of 0.047 is required for livestock to transition between standing and lying based on behavioural and kinematic observations performed on cattle (7). Australian live exported sheep are currently housed at a *k-*value of 0.03 from November to April, and 0.033 from May to October (8), but shipments to the Middle East are prohibited from departing between 1 June and 14 September because of increased heat stress risk for sheep during this period (9). Research to date has not compared the implications of the specific *k*-values for sheep welfare or other values within that range. Although there has been some research to determine the optimal stocking density, previous stocking density studies have failed to assess a broad range of validated welfare indicators, largely relying on productivity measures and limited behavioural outcomes, and often fail to account for differences in group size (10, 11), which appears regularly as a confounding variable in stocking density research.

Alongside the quantity of space provided, the quality of space also impacts animal behaviour and welfare through the provision of various resources within a pen environment (3, 12). Feed, in particular, is a resource that animals in high-density housing may have to compete either because of the space available to access it or the quantity of feed itself (13). The quantity of feed provided during voyages is regulated at less than *ad libitum* allowance, but feed trough space on-board vessels is not required to permit simultaneous feeding by all animals in a pen (14), potentially increasing competition (15) and reducing the quality of space for some sheep. Furthermore, the quality of available space may interact with stocking density for welfare outcomes of animals (16).

The aim of this study was to assess the welfare implications for sheep of live weight W housed at five stocking densities varied by the *k-*value in the allometric space equation area = *k*W^2/3^, with either unrestricted or restricted trough allowance. In this first of a series of related experiments, we observe the effect of stocking density and trough space with few additional stressors (i.e., thermoneutral conditions and without ship movement). In each experiment, we include accumulative stressors to unpack the impact of various environmental factors sheep may experience during live export and how (or if) stocking density is related. We anticipated that reducing space allowance would lead to reduced sheep welfare, as indicated by various physiological and behavioural assessments such as potential increases in FGCM concentrations and changes in important lying behaviours, and that the impact of reduced space would be exacerbated when trough space was also restricted.

## Materials and methods

The experiment was undertaken at The Commonwealth Scientific and Industrial Research Organisation (CSIRO) McMaster Laboratory, Armidale, New South Wales (NSW), Australia. The conduct of the experiment was approved by the CSIRO Chiswick Animal Ethics Committee under the NSW Animal Research Act 1985 (approval ARA 20/05).

### Experimental design

The experimental design was a randomised block experiment with 10 pen-level treatment combinations allocated in a factorial design of five levels of stocking density *k-*value using the allometric space equation area = *k*W^2/3^, and two levels of feed trough space implemented in two time periods (runs), each with 20 pens (two of each of the 10 treatment combinations; Table 1). Within each run, for each of the 10 treatment combinations, lower initial live weight category sheep were allocated to one pen and higher live weight category sheep were allocated to the other pen. The sheep were housed in their treatment pens for 18 days, which is comparable to relevant live export voyage lengths (17).

**Table 1:** Randomised block experimental design for stocking densities and trough space restriction treatment groups.

**Stocking density *k-*value^a^**	**Area (m^2^ per 40.6 kg sheep)**	**Trough space**	
		**Restricted 6 cm/head**	**Unrestricted 16 cm/head**
0.027	0.31	18 head x 2 pens x 2 runs	18 head x 2 pens x 2 runs
0.032	0.37	18 head x 2 pens x 2 runs	18 head x 2 pens x 2 runs
0.037	0.43	18 head x 2 pens x 2 runs	18 head x 2 pens x 2 runs
0.042	0.48	18 head x 2 pens x 2 runs	18 head x 2 pens x 2 runs
0.047	0.54	18 head x 2 pens x 2 runs	18 head x 2 pens x 2 runs

^*a*^area = kW^2/3^ (4).

### Animals and induction protocol

For each run, 375 recently shorn Merino wethers (initial mean live weight ± SD 40.9 ± 4.1 kg and 40.2 ± 6.2 kg for runs 1 and 2, respectively) between 2- and 4-tooth were trucked to the experiment location and inducted into the animal house. Merino wethers represent the most common class of sheep transported by sea from Australia (18).

On arrival, the sheep were weighed, body condition scored, given an oral anthelmintic (Startect^®^, Zoetis Australia, Silverwater, NSW), and ear tagged for individual identification. To adapt the sheep to the feed and facilities before the treatments were applied, they were housed in large group pens at a stocking density *k-*value of 0.05 for 14 and 7 days for runs 1 and 2, respectively, permitting them more space than any of the treatment stocking densities. Each group pen contained a large automatic water trough and metal feed troughs (14 cm trough space per head). During the adaptation period, the sheep were introduced to a commercial dietary shipper pellet (9.5 MJ of ME and 12.1% CP per kg of DM; Macco Feeds, Willams, Western Australia). The sheep were fed with a mix of 50% oaten chaff and 50% pellets for the first day of adaptation. The amount of chaff was reduced to 25 and 15% for days 2 to 3 and 4, respectively. From day 5 of adaptation onwards and during the experimental period, the sheep were fed with pellets exclusively. The feed allowance for the entirety of the trial was calculated at 2.85% of live weight as feed dry matter, offered in two meals per day at 08:00 and 14:30 h.

### Treatment allocation

For the first run, the sheep arrived at the animal house in five cohorts originating from five farms, (*n* = 2, 9, 20, 60, and 284 animals from cohorts 1–5, respectively). Cohort 2 (*n* = 9) was maintained as spare sheep and kept separately in the animal house for the duration of the acclimation period, after which they were no longer required for the experiment. Two sheep from cohort 5, two sheep from cohort 3, and two sheep from cohort 4 were excluded for health reasons. The remaining 360 sheep were weighed 3 days before the commencement of the experimental period and stratified by live weight into two blocks, heavy (*n* = 180, range = 44.5–62 kg) and light (*n* = 180, range = 33–44.5 kg).

Within each live weight block and cohort, the sheep were randomly allocated to one of the 10 groups, each containing 18 sheep. This ensured that each cohort provided the same proportion of sheep within each group as far as possible. The groups were then randomly allocated to the 10 treatments within live weight blocks. Three animals from each pen were randomly allocated as a focal sheep. The selection of three focal animals was based on a power analysis using relevant data (19) to determine a suitable sample size for detecting changes in the most variable physiological parameter, FGCM concentrations.

For the second run, the sheep arrived in four cohorts originating from four properties (*n* = 3, 7, 20, and 346 animals from cohorts 1, 2, 3, and 4, respectively). Cohorts 1 and 2 were kept aside as spare animals. Four sheep from cohort 4 were removed because of health reasons, and two more from this cohort were removed to normalise the live weight distribution. The remaining 360 sheep were stratified and allocated to heavy (*n* = 180, range = 39.5–57 kg) and light (*n* = 180, range = 26.5–40 kg) weight blocks, groups, treatments, and as focal animals in the same manner as for run 1.

### Treatment pen design

The pens were in a raised, fully enclosed shed with metal grated flooring through which urine and faecal matter could fall through gaps approximately 1 × 2 cm in size. The pen area was adjusted by moving the panels to maintain allocated space within ±0.001 m^2^ of the area required by the allometric equation, area = *k*W^2/3^ (4), where *k* is the treatment *k-*value and W is the mean live weight of the 18 animals in the pen. The area was then multiplied by 18 to correspond to 18 head in each pen. All the treatment pens were randomly located within the animal house, and location was re-randomised for run 2. During the second run of the experiment, one animal was removed on day 10 because of an injury, and the pen space was adjusted immediately to maintain the correct stocking density.

At each stocking density, feed troughs were provided at either 6 or 16 cm per head for restricted and unrestricted treatment groups, respectively. The provision of 6 cm per head is comparable to the maximum head space provided during voyages, as calculated on a live export vessel (based on 10 pen trough length measurements in 2020, AT, per comm.). The provision of 16 cm reflects the recommended trough space for sheep being housed in scientific institutions, and aims to eliminate competition for feeding space (20). Suspended metal feed troughs were hung on pen rails between 50 and 70 cm above the pen floor. A wooden plank was fastened beneath the troughs, ensuring that the space underneath was unusable by the wethers. Feed was provided to the sheep in a random order that differed each day and feeding time. Unrestricted water access was provided in a minimum of four hanging plastic troughs ranging from 12 to 18 L in capacity. The wethers always had access to clean water, and troughs were cleaned and refilled once or twice daily as required. Total water trough length was provided at 9.3 and 12.4 cm per head for restricted and unrestricted trough treatment pens, respectively.

The natural photoperiod at the start of the trial occurred for approximately 12 h between 06:00 and 18:00 h. The natural photoperiod had increased to approximately 14 h between 06:00 and 20:00 by the end of the second run. Artificial lighting was only used between 06:00 and 07:00 h on days 6, 12, and 18, as natural light levels were too low to safely conduct faecal sampling.

### Environmental monitoring

Two Kestrel D3 Fire Drops (Nielsen-Kellerman, PA, United States) were suspended from the ceiling in opposite corners of the shed that continuously logged temperature and humidity data at 10-min intervals.

The average dry bulb temperature (T_DB_) over all time points over 18 days was 16.6 and 19.4°C for runs 1 and 2, respectively, and the average relative humidity (RH) over all time points over the 18 days for these runs was 67 and 61.8%, respectively. The average wet bulb temperature (T_WB_) over all time points over 18 days was 12.5 and 14°C for runs 1 and 2, respectively, with a slight variation observed between runs because of seasonal changes in ambient conditions. Supplementary Table S1 summarises the mean T_DB_ and T_WB_, as well as RH, for both runs.

### Behavioural analysis

Video footage was continuously recorded with fixed infrared cameras (MR6822E2 and LR832; Lilin Australia Pty Ltd., Lidcombe, NSW) positioned above each individual pen for the entire duration of the experimental period. The behavioural analysis consisted of continuous analysis of focal animals as well as scan sampling for all animals for standing and lying position behaviours.

Continuous behavioural observations were conducted using the same three focal animals in each treatment pen for two 5-min periods on days 3, 5, 11, and 17. The active observation period (10:25–10:30 a.m.) and inactive observation period (12:25–12:30 p.m.) were selected to represent times of day when sheep were typically active and moving around their pen or in a more restful state, respectively, as determined by preliminary scan sampling of all pens over all hours on days 3, 5, and 11. The video footage used for analysis was not labelled with the treatment *k-*value stocking density, but visual differences in space allowances and trough space restrictions meant the two observers were not entirely blind to the treatment groups during data collection. Focal sheep behaviour was categorised according to the definitions outlined in Table 2 using the Observer XT© 15.0 software (Noldus Ltd., Wageningen, The Netherlands). Agonistic interactions and displacement events were analysed as count of observations due to their very short durations. Other behaviours were scored as state behaviours, with a start and end time for each bout so that total durations could be calculated for both observation periods.

**Table 2:** Ethogram for categorising focal sheep behaviours during continuous observation periods.

**Behaviour**	**Definition**
Lying with head down	Sternum and/or sideline in contact with floor. Head is resting on the floor, part of theirown body or on any part of a conspecific. Bout complete when head becomes elevated for more than 2 s or sternum or sideline is no longer in contact with floor.
Lying with head up	Sternum and/or sideline in contact with floor. Head is lifted. Bout complete when head is placed down for more than 2 s, or sternum or sideline is no longer in contact with floor.
Standing with head down	All four hooves in contact with floor, sheep stationary for longer than 2 s. Head is held parallel to shoulder or lower. Bout complete when the sheep begins to walk or lie or head moves higher than the shoulder for more than 2 s.
Standing with head up	As above, with head held above shoulder height for more than 2 s.
Eating	Sheep has head directly over the feed trough when pellets are present and can be observed. Bout complete when moved back from trough for at least 3 s.
Drinking	Head directly over water trough. Bout complete when moved back from drinking trough for 3 s.
Interaction with environment	Muzzle used to touch: pen environment (fence panel, gate), pen floor, edge or handle of water trough (not consuming water), feed trough (head over feed trough but no *unsoiled* pellets are present. Assumed any feed pellets that remain after 90 min of feed being provided to that pen were soiled). Bout complete when contact has ceased for 3 s or more.
Grooming self	Sheep muzzle or hoof comes in contact with another part of their own body and is used to scratch or rub. Includes using aspects of the facility to scratch one's self.
Grooming conspecific	Sheep muzzle comes in contact with a conspecifics body and is used to scratch or rub in a back-and-forth motion.
Locomotion	Two steps in any direction, without stopping for more than 2 s. Bout complete if animal is stationary for more than 2 s.
Nesting	Sheep pawing at ground. Bout complete when sheephas stopped pawing for more than 2 s. Considered a successful bout if the sheep lies down following nesting, or unsuccessful if they remain standing.
Unknown	Sheep not visible in the image or visible but behaviour is unknown.
Agonistic displacement	A sheep uses agonistic behaviours to move a conspecific and take its place. Possible agonistic behaviours include head-butting, mounting or pawing. The sheep for which the behaviour is scored can be the initiator or recipient of the displacement.
Agonistic without displacement	A sheep directs agonistic behaviours at a conspecific without displacing it. Possible agonistic behaviours include head-butting, mounting or pawing. The sheep for which the behaviour is scored can be the initiator or recipient of the agonistic behaviour. Each head butt, mounting, or pawing (single paw) attempt (making contact with the conspecific or not) is recorded as an occurrence.
Non-agonistic displacement	Physical contact (such as bumping, brushing past) excluding agonistic behaviours outlined above, followed by taking place of affected conspecific. Initiating sheep may or may not continue their locomotion. The sheep for which the behaviour is scored can be the initiator or recipient of the displacement.

One observer (BM) with prior experience in quantifying sheep behaviour (21) annotated the continuous focal videos. The intra-observer reliability was assessed by re-scoring eight randomly selected video pairs (four pairs from each active or inactive observation period) in two different stages (at the start and end of the continuous behavioural data collection period) and had an average kappa value of 0.71 among the eight video pairs tested, ranging between 0.58 and 1. This indicated that while there was some variation in intra-observer reliability between different pairs of videos, the average level of reliability is acceptable when interpreted according to Martin and Bateson (22).

Scan sampling of positional behaviour was performed for all the animals in each pen at hourly time points (using still images at these time points) over the 24-h period on days 3, 5, 11, and 17 excluding five time points on each day during which the animals were disturbed because of human presence (08:00, 09:00, 10:00, 14:00, and 15:00 h). The scan sampling interval of 60 min was determined by conducting a preliminary scan sampling analysis in which observations were made every 15 min, and then comparing the representation of behaviours using data every 15, 30, 45, and 60 min at three different *k-*value stocking densities. The behaviours scored during the scan sampling analysis are described in Table 3, and these data were collected in Microsoft Excel [Microsoft Corporation (2016) https://office.micrsoft.com/excel].

**Table 3:** Ethogram utilised to record positional behaviours for all the sheep at scan sampling observation times.

**Position**	**Definition**
Standing	Sheep is upright with at least three hooves (so could be performing locomotion) in contact with the pen floor
Lying 1	All 4 legs kept close to body. There is no gap between the lower portion of a back leg and the body. No front legs are stretched out
Lying 2	All 4 legs sticking out away from body
Lying 3	1 or 2 back legs sticking out from body with both front legs close to body OR 1 or 2 front legs stretched out from body with both back legs close to body
Lying 4	At least 1 back leg and one front leg stretched out from body (bottom half of leg not touching body of sheep)
Lying unknown	Cannot see enough legs to determine position
Body 1	Sheep body or limb in direct contact with another sheep
Body 2	Sheep is not touching another sheep with its body or limb
Body unknown	Cannot determine if sheep is touching another sheep
Head 1	Head is held up
Head 2	Head is down and resting, placed on the floor, the pen, or its own body
Head 3	Head is down and resting, placed on another sheep
Head unknown	Cannot determine the head position of the sheep

One observer (MA) was trained by BM to perform the scan sampling analysis. The training consisted of both observers applying the ethogram (Table 3) to approximately 20 images until MA was confident in applying it independently. The training continued until values of 0.8 were achieved for each of Lin's concordance correlation and intraclass correlation coefficients for agreement in counts for the trainer (BM) and the trainee (MA) among 56 time point images (refer to Supplementary Table S2). Agreement was not assessed for lying 2 because of the rarity of this behaviour in the test data. MA maintained a high ability to score behaviours according to the initial training over time, with agreement for most behaviours exceeding the acceptability threshold of 0.7 at the conclusion of the data collection period (refer to Supplementary Table S2) (22). Intra-observer reliability was assessed at the end of the data collection period by asking MA to reanalyse a subset of 57 unmarked images that had been analysed in different stages of the data collection process (i.e., immediately after training, when half of the data were collected, and when the dataset was complete). Lin's concordance correlation and intraclass correlation coefficients were calculated and indicated an acceptable level of reliability (>0.7) (22) (refer to Supplementary Table S3).

### Physiological sampling

All physiological sampling was performed in the morning before feed was provided and completed within 60 min; individual focal sheep were restrained in their pens for approximately 3 min during sample collection. Baseline blood samples were collected from all the focal animals on days −9 and 0 for run 1, and days −2 and 0 for run 2; the mean of the two measures was used for analysis. Additional blood samples were collected at the end of each run on day 18. The blood samples were taken by jugular venipuncture and collected in 10-ml K_2_-EDTA vacutainer tubes (BD, Lane Cove, NSW, Australia). Commercially available clippers were used to remove wool along the jugular region where required. The blood samples were stored at 4°C for approximately 1 h before being placed in a rotary tube mixer for 4 min. Immediately after mixing, total leucocytes, neutrophils, and lymphocytes were quantified with an Abbott-Cell-Dyn Counter 3700 (Abbott Diagnostic Division, Vienna, Austria).

**Figure 1:**
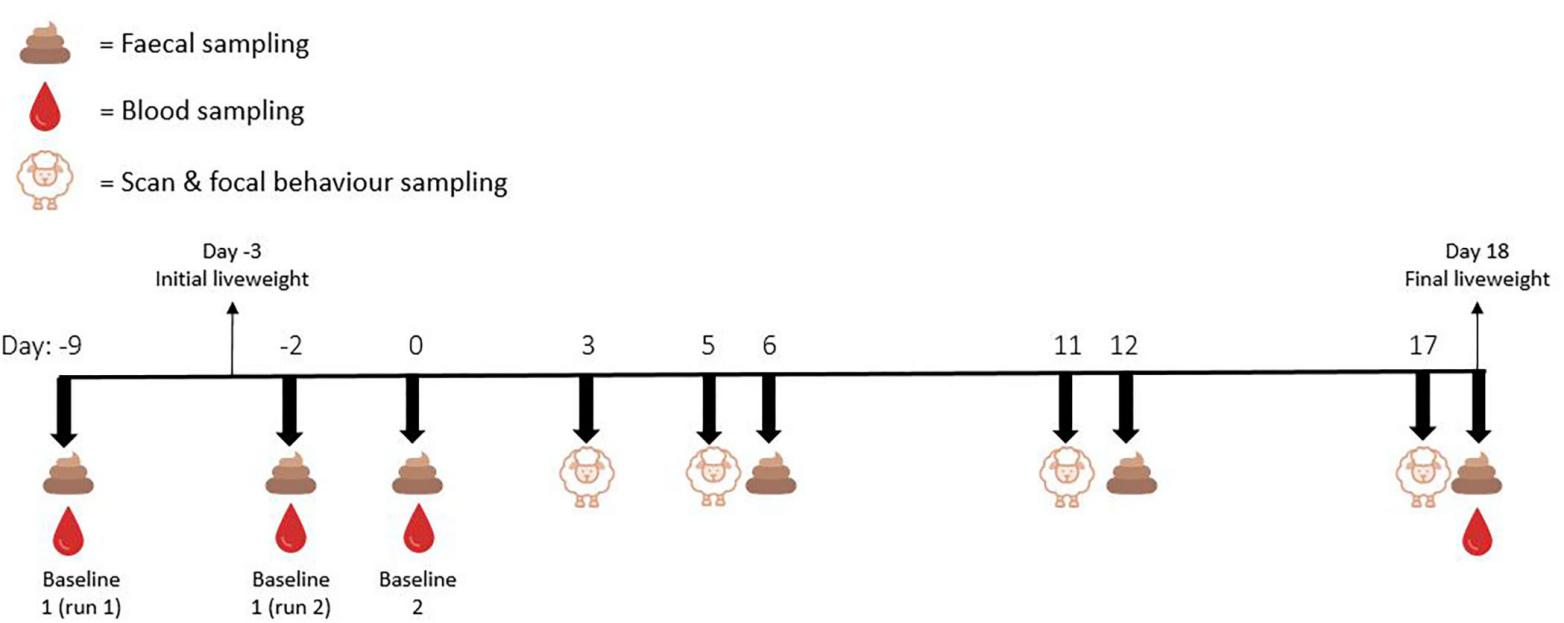
Experimental timeline depicting the days on which blood, faeces, whole of pen scan sampling behaviours, and focal animal continuous behaviours were sampled.

Baseline faecal samples were collected from focal animals on day 0 for both runs 1 and 2, and additional faecal samples were collected on days 6, 12, and 18. Up to six faecal pellets were taken directly from the rectal ampulla of each focal animal before being placed immediately on ice. Figure 1 represents a visual depiction of the experimental timeline.

The faecal samples were frozen within 40 min after collection and stored at−20°C until processing. The samples were oven-dried at 60°C for 48 h and ground using a ceramic hand grinder. The individual samples were then analysed using a method that has also been applied to cattle (23). During validation of this method, the efficiency of extraction was 86 ± 3%, calculated using five samples spiked with cold cortisol. The repeatability of this assay result was 96% as calculated on six repeats of four individual samples. The parallelism of the assay was checked by serial dilution of three individual extracts. For the current methodology, 100 mg of dried sample was reconstituted in 300 μl of double distilled water followed by vortex for 5 min. This was added to 2,700 ml of 100% ethanol, vortexed for 10 min, and then spun at 2,000 G for 10 min. The supernatant was decanted in glass tubes. Pellets were extracted again with 3 ml of 100% ethanol and spun at 2,000 G for 10 min. The supernatant was added to the previous extract. The extracts were dried under airflow for 5 to 6 h and then were reconstituted in 500 μl of phosphate buffer saline (pH 7.4), vortexed for 10 min, and spun at 1,000 G for 2 min. Extraction efficiency was 86 ± 3%. Concentrations of faecal cortisol metabolites (FCMs) in the extract were measured in duplicate using the MP Biomedical I125 RIA Cortisol Kit (# 07-221106; MP Biomedicals Australia, Seven Hills, NSW). The limit of detection was 0.2ng/ml and the mean inter-assay coefficients of variation were 6.5% (1.4 ng/ml) and 1.5% (3.2 ng/ml). The results are expressed as nanograms of FGCM per gramme of dry faeces.

### Statistical analyses

Statistical analyses were performed in Stata (StataCorp, Release 16. College Station, TX). Trough space, *k*-value (for dependent variables measured on days 3, 5, 11, and 17), and day, were simultaneously fitted as fixed effects in regression models. Trough space allowance was analysed as unrestricted compared to restricted, and *k*-value was fitted as a continuous variable. Day was fitted as a continuous variable for most models. There was reasonable evidence of non-linearity for the scan sampling data, so day was fitted as a categorical variable in these instances. Fractional polynomial terms for *k-*value and day were included as a continuous variable and were considered for statistical models of all outcome variables to assess for non-linearity of relationships. Stata's -fp- command was used, and all possible one- and two-dimension polynomials for powers of −2, −1, −0.5, 0, 0.5, 1, 2, and 3 (44 models in total) were assessed. For all outcome variables, there was no strong evidence for non-linear relationships for either *k-*value or day number, so any relationships between these and the outcome variables were assumed to be close to linear. Interactions among *k*-value, trough space, and day (where fitted) were assessed. No three-way interactions were retained in final models because the associated coefficients were biologically implausible. Effects were considered to occur based on joint consideration of views of the prior evidence for the association in combination with the *p*-value (24).

Mixed effects linear regression was conducted to assess the effects of *k-*value and trough space allowance on day 18 live weight, concentrations of leucocytes, lymphocytes, and neutrophils in blood on day 18, and neutrophil to lymphocyte ratio (log_e_-transformed) on day 18. Individual sheep was the unit of analysis and sheep group was fitted as a random effect. The baseline value for each sheep was fitted as a covariate. Neutrophil to lymphocyte ratio data were log-transformed prior to analyses. A repeated measures mixed effects linear regression model was used for analysing FGCM concentration over time, with time point within sheep as the unit of analysis, sheep fitted as a random effect, an unstructured residual correlation matrix used, and time point number included as a fixed effect.

Counts of each of agonistic and displacement events summed for the three focal sheep in each group for each observation period were analysed by negative binomial regression, for each of the 10:25 a.m. and 12:25 p.m. 5-min observation periods separately; observation period within sheep group was the unit of analysis, and group was included as a random effect. The random effect of group was omitted if the model with that random effect failed to converge.

Proportions of the total sheep time in which the three focal sheep were observed in each 5-min observation period (a total of 900 sheep s when each focal sheep was visible on video for the entire 5 min) that a focal animal spent exhibiting various state behaviours were analysed for the 10:25 a.m. and 12:25 p.m. observation periods separately. Generalised linear models were used with observation period within sheep group as the unit of analysis, binomial residual distribution, logit link function, and robust standard errors that accounted for clustering of observation period within group. Model choice was limited by the fact that there were a high number of zero proportions in the dataset, and a generalised linear model was determined as most suitable despite not accounting for serial correlation across days.

Pooled numbers of behavioural transitions performed by the three focal animals during each observation period were also analysed with observation period within sheep group as the unit of analysis. The number of transitions between all state behaviours was analysed, as well as the number of transitions between standing behaviours and lying. All state behaviours except lying were assumed to be standing behaviours in this context. Negative binomial regression was conducted with sheep group included as a random effect.

Kappa coefficients for the synchronisation of lying were calculated for each 24-h period for each group on days 3, 5, 11, and 17 (40 groups by 4 days = 160 κ coefficients) using numbers of sheep lying at each of the hourly scan sampling time points taken at 19 time points within the 24-h periods according to the methods outlined in Rook and Penning (25). Briefly, the kappa coefficients of agreement were calculated based on the number of sheep lying at each time point, and the proportion of synchronisation at each time was determined by comparing the number of pairs of sheep lying to the total number of possible pairs (25). The kappa coefficient is 1 only if at each time point within the 24-h, all the sheep have the same lying status, e.g., at some time points, all the sheep are lying; at other time points, all the sheep are not lying. A kappa coefficient of 0 indicates that the number of sheep lying within time points in the 24-h period was no greater than that expected by chance. Kappa coefficients were analysed using mixed-effects linear regression models with group included as a random effect, and a first order autoregressive residual correlation structure was used.

For other scan sampling data, the unit of analysis was the hourly time point within sheep group. Diurnal patterns in proportions of groups exhibiting standing and lying behaviours were evident, so trigonometric (sine and cosine) predictors (26) with one complete period per 24 h were included to account for time of day effects for all the scan sampling behaviour models. Numbers of animals standing at each hourly time point were analysed by zero-inflated negative binomial regression with robust standard errors that accounted for clustering of observation period within group. For numbers of other positional behaviours, mixed effects generalised linear models regression was conducted with group included as a random effect. For all the models, the number of animals eligible to exhibit the behaviour was included in the model to account for the amount of exposure from which the behaviours were observed. Thus, for analyses of numbers of animals standing, the amount of exposure was the total number of sheep in the group at the hourly time point, and for analyses of number of animals lying in body contact with another sheep, the amount of exposure was the number of sheep in the group that were lying at the hourly time point. The same exposure was used for analyses of number of lying sheep with head resting (head positions 2 and 3 pooled; Table 3) and analyses of number of lying sheep with one or more legs outstretched from their body (lying positions 2, 3, and 4 pooled; Table 3). For analyses of number of lying sheep placing their head on a conspecific (head position 3; Table 3), the amount of exposure was the number of sheep lying with their head down. Exposure counts did not include sheep on which the behaviour could not be observed on the video, and observation periods with no sheep exposed were excluded from analysis of that particular dependent variable.

In interpreting our results, we implement the principles listed in the American Statistical Association 2016 statement on statistical significance and *p-*values (27) and the methods described by Rothman and Lash (28). Thus, while we refer to *p-*values as being significant or not significant, we have also considered alternative hypotheses values that, based on our confidence intervals, are not compatible with our data. We have also considered our prior views about the probability that the null hypothesis is the truth in the target population when interpreting *p-*values as recommended by Goodman (29). As such, our description of effects as “important” is not based on statistical significance alone but also the size of any potential effects in the context of real implications for sheep, as indicated by confidence intervals.

## Results

### Scan sampling behaviour

Kappa values for the synchronicity of lying (calculated for each 24-h period for each group) ranged from 0.11 to 0.37, and the *p-*value for the effect of *k-*value on kappa was 0.076. The largest effects consistent with these results (based on 95% CI limits) were small and would be unlikely to have any biological importance (estimated difference in mean kappa for each 0.01 increase in *k*-value = 0.011; 95% CI – 0.001 to 0.024). There was no important effect observed of trough space allowance on lying synchronicity (estimated difference in mean kappa for unrestricted compared to restricted troughs = 0; 95% CI −0.01 to 0.02; *p* = 0.694).

For analyses of numbers of animals standing at each of the 19 observation time points within the 24-h period, from the count component of the model, the estimated ratio of proportions of sheep that were standing was 1 for every 0.01 increase in *k*-value (95% CI 0.93–1.07, *p* = 0.968) and 1.01 for restricted trough space (relative to unrestricted trough space; 95% CI 0.92–1.1, *p* = 0.874). From the inflate part of the model (the component modelling whether there was an excess number of time points when no sheep were standing over that expected from a standard negative binomial model), the estimated ratio of the odds of no sheep standing was 1.05 for every 0.01 increase in *k*-value (95% CI 0.93–1.18, *p* =0.419) and 1.01 for restricted trough space (relative to unrestricted trough space; 95% CI 0.94–1.29, *p* = 0.24).

Increased *k-*value (i.e., more pen space) decreased the proportion of lying animals that were in physical body contact with a conspecific. The proportion was estimated as changing by a factor of 0.94 for every 0.01 increase in *k*-value (95% CI 0.93–0.95, *p* < 0.001). Thus, the proportion of lying sheep in physical body contact with a conspecific at *k* = 0.037 was estimated to be 0.94 of (i.e., lower than) that at *k* of 0.027, and at *k* = 0.047, it was estimated to be 0.94^*^0.94 or 0.88 of that at *k* = 0.027. The mean proportions and standard deviations of lying sheep in physical body contact with a conspecific across all time points for *k*-values 0.027–0.047 was 0.8 (±0.33), 0.82 (±0.31), 0.79 (±0.32), 0.75 (±0.32), and 0.72 (±0.32), respectively. Trough space had no important effect on the proportion of lying animals that were in physical body contact with a conspecific (estimated change in proportion for unrestricted troughs compared to restricted = 1.01; 95% CI 1–1.03; *p* = 0.128).

**Figure 2:**
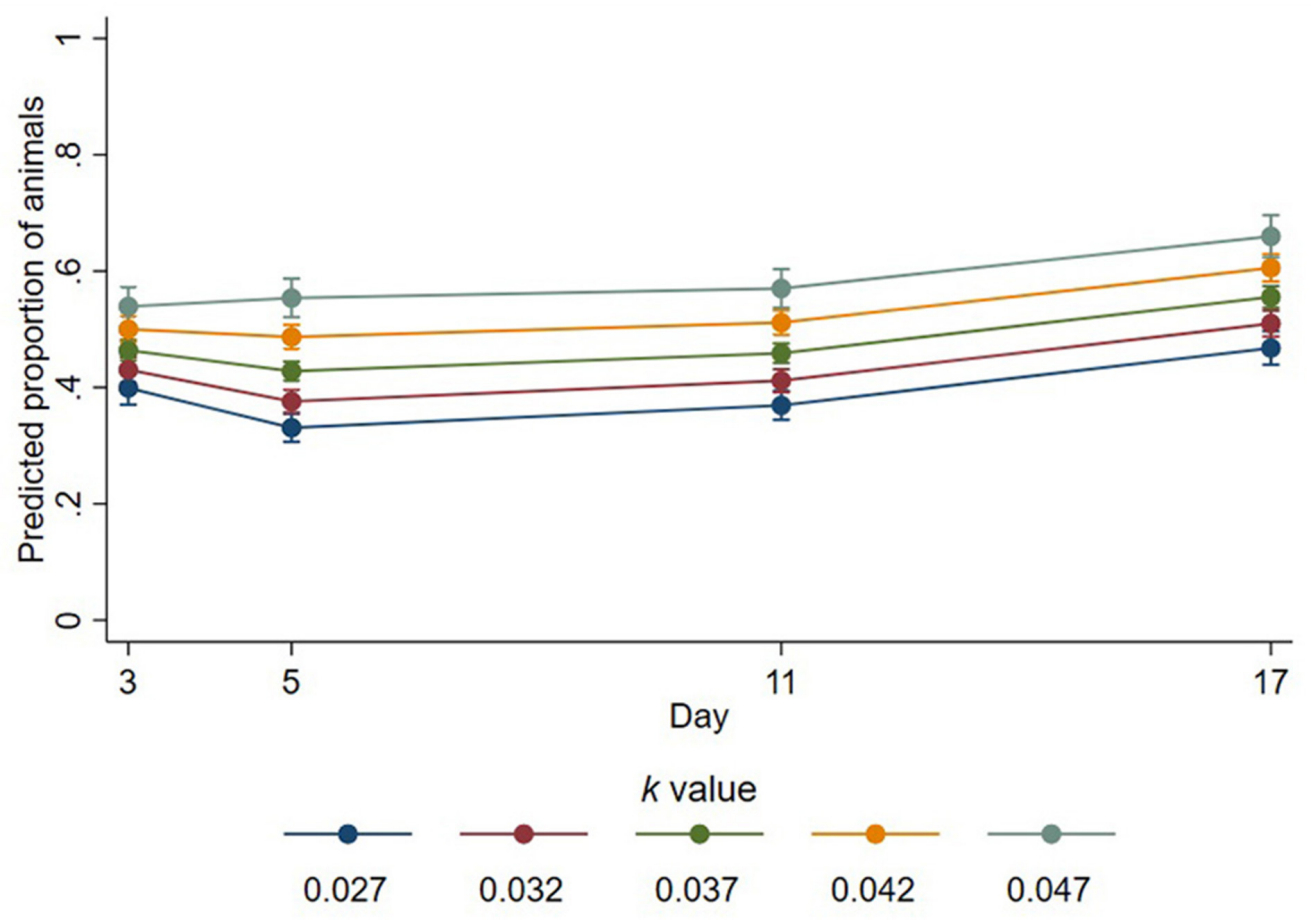
Predicted proportions of lying animals that had outstretched legs at different *k*-values across days. Error bars represent 95% confidence intervals of predicted proportions. Predicted proportions were calculated as predicted numbers divided by the average number of sheep lying at each time point (12.48).

Of the sheep that were lying, the proportion lying with legs outstretched was affected by a significant interaction between *k-*value and day (*p* = 0.001, Figure 2). On day 3, the proportion of sheep with legs outstretched increased with *k*-values, estimated as increasing by a factor of 1.16 for every 0.01 increase in *k*-value (95% CI 1.1–1.23, *p* < 0.001). The proportions of sheep lying with legs outstretched also increased on days 5, 11, and 17 by estimated factors of 1.29 (95% CI 1.23–1.37, *p* < 0.001), 1.24 (95% CI 1.18–1.31, *p* < 0.001), and 1.19 (95% CI 1.13–1.25, *p* < 0.001) for every 0.01 increase in *k-*value, respectively. The mean proportions and standard deviations of lying sheep with outstretched legs across all time points for *k*-values 0.027–0.047 were 0.33 (±0.23), 0.43 (±0.26), 0.45 (±0.25), 0.49 (±0.26), and 0.52 (±0.26), respectively. Trough space had no important effect on the proportion of animals lying with outstretched legs (estimated change in proportion for unrestricted troughs compared to restricted = 1.03; 95% CI 0.97–1.08; *p* = 0.363).

*k-*value had a significant effect on the number of lying animals that were supporting their head either on themselves, on a part of the pen, or on a conspecific. Of those lying, the proportion of sheep supporting their head was decreased with increases in *k*-value, estimated as changing by a factor of 0.82 for every 0.01 increase (i.e., more space) in *k*-value (95% CI 0.79–0.85, *p* < 0.001). Trough space allowance did not significantly affect the proportion of lying animals that had their heads down (estimated change in proportion 0.99; 95% CI 0.94–1.04; *p* = 0.602).

**Figure 3:**
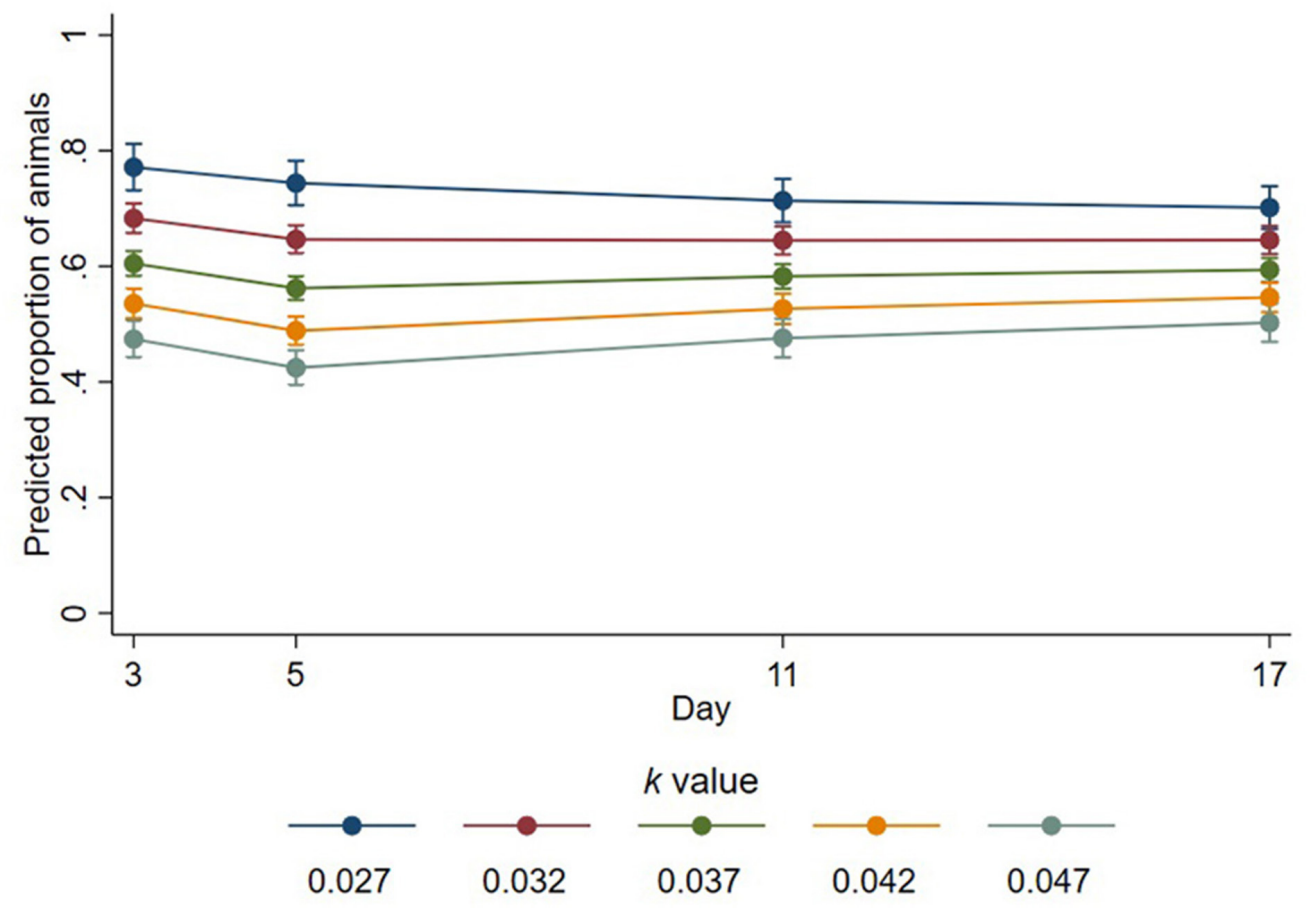
Predicted proportions of animals lying with their head down that place their head on a conspecific at different *k*-values across days. Error bars represent 95% confidence intervals of predicted proportions. Predicted proportions were calculates as predicted numbers divided by the average number of sheep lying at each time point (6.69).

Of the sheep that were lying with their head down on themselves, on a part of the pen, or on a conspecific, the number of those resting their head on a conspecific was determined by interaction between *k-*value and day (*p* < 0.001, Figure 3). On day 3, of those lying with their head down, the proportion of sheep resting their head on a conspecific was estimated as decreasing by a factor of 0.78 for every 0.01 increase in *k*-value (95% CI 0.75–0.82, *p* < 0.001). The proportion of those resting their head on a conspecific was decreased on days 5, 11, and 17 by estimated factors of 0.76 (95% CI 0.72–0.79, *p* < 0.001),0.83 (95% CI 0.78–0.86, *p* < 0.001), and 0.85 (95% CI 0.81–0.89, *p* < 0.001) for every 0.01 increase in *k*-value, respectively. There was no significant effect of trough space allowance observed over all days pooled (estimated change in proportion = 1.01; 95% CI 0.95–1.06; *p* = 0.801).

### Focal animal continuous behaviour

**Figure 4:**
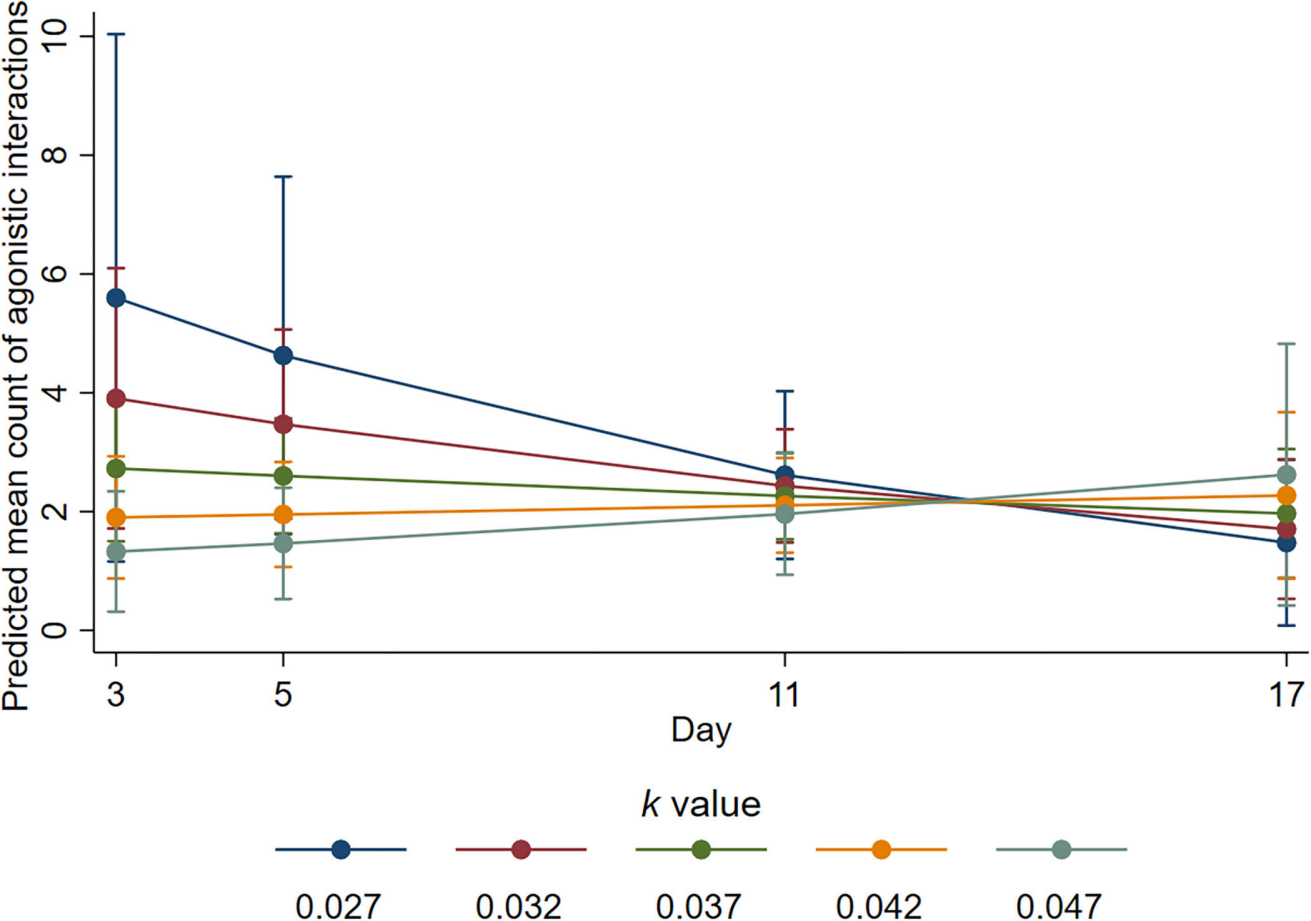
Predicted mean counts of agonistic interactions during the active observation for all *k*-value treatments on each observation day. Error bars represent 95% confidence intervals of predicted mean numbers.

During the active observation period, there was evidence of statistical interaction between *k-*value and day for the number of agonistic interactions (*p* = 0.064). On day 3, the mean number of agonistic interactions was decreased by a factor of 0.49 with every 0.01 increase in *k*-value (95% CI 0.26–0.92, *p* = 0.026), and on day 5, the mean number of agonistic interactions was decreased by a factor of 0.56 for every 0.01 increase in *k-*value (95% CI 0.33–0.95, *p* = 0.032). However, on day 11, the estimated ratio of mean counts was 0.87 (95% CI 0.57–1.32, *p* = 0.502), and on day 17, it was 1.33 (95% CI 0.66 to 2.7, *p* = 0.425). On day 3, the fitted mean number of agonistic interactions was highest at *k*-value of 0.027 (i.e., least space), but for this *k*-value, mean numbers were decreased from day 3 such that by day 11, mean numbers were similar for all *k*-values (Figure 4).

**Figure 5:**
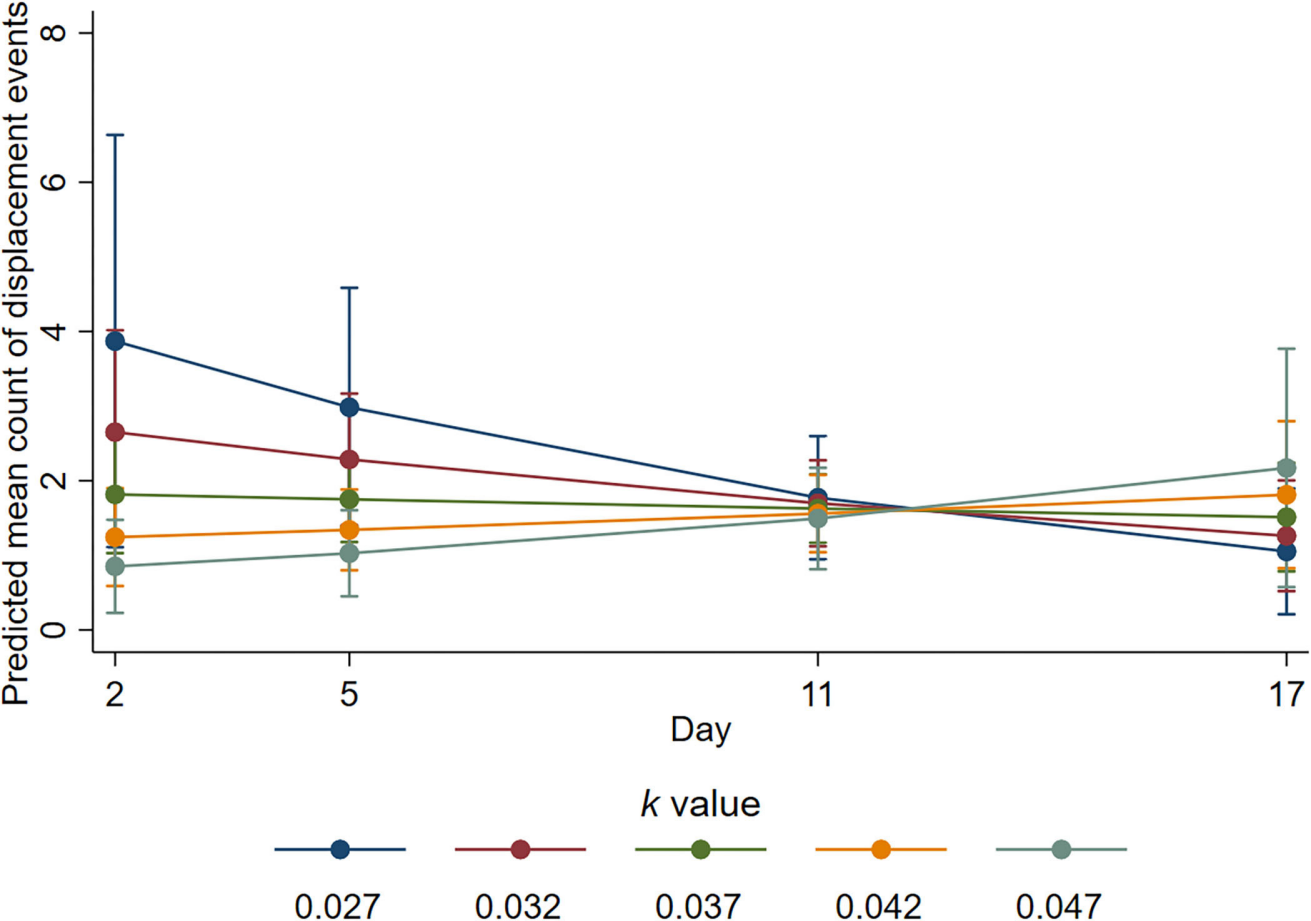
Predicted mean counts of displacement events during the active observation for all *k*-value treatments on each observation day. Error bars represent 95% confidence intervals of predicted mean numbers.

There was also evidence of an interaction between *k-*value and day for the number of displacement events during the active observation (*p* = 0.02). The mean number of displacement events was estimated as decreasing by a factor of 0.51 on day 3 (95% CI 0.3–0.86, *p* = 0.011) and by a factor of 0.58 on day 5 (95% CI 0.38–0.91, *p* = 0.018), for a 0.01 increase in *k*-value (i.e., more space). However, the estimated ratio of mean counts on day 11 was 0.92 (95% CI 0.64–0.32, *p* = 0.636); on day 17 it was 1.44 (95% CI 0.79–2.62, *p* = 0.236). On day 3, the mean number of displacement interactions was highest at *k*-value of 0.027 and least at *k*-value of 0.047, but for these *k*-values, respectively, mean numbers decreased and increased from day 3 such that by day 11, the mean numbers were similar for all *k*-values (Figure 5).

Trough space allowance did not significantly affect counts of agonistic (estimated ratio of mean counts = 1.35; 95% CI 0.74–2.46; *p*= 0.329) or displacement events (estimated ratio of mean counts = 1.36; 95% CI 0.8–2.32; *p* = 0.25) in the active observation. During the inactive observation, no important effect of trough space was observed for agonistic (estimated ratio of mean counts = 0.81; 95% CI 0.39–1.69; *p* = 0.576) or displacement (estimated ratio of mean counts = 0.95; 95% CI 0.48–1.86; *p* = 0.871).

The proportion of time that the focal animals spent exhibiting some state behaviours during the active and inactive observation periods are shown in Table 4.

**Table 4:** Proportion of time the focal animals spent exhibiting selected state behaviours during continuous observation periods (mean ± SD).

**Behaviour**	**Active period**	**Inactive period**
Lying	0.58 ± 0.37	0.68 ± 0.37
Standing	0.31 ± 0.29	0.24 ± 0.28
Grooming	0.01 ± 0.02	0.01 ± 0.03
Locomotion	0.02 ± 0.02	0.01 ±0.02
Interacting with environment or conspecific	0.04 ± 0.05	0.04 ± 0.08

The generalised linear model results for the continuous analysis of focal animal behaviour during the active and inactive observation periods are shown in Table 5. The results are presented as the ratio of the odds of all three focal animals spending 100% of the observation time engaged in behaviour compared to the odds of all three focal animals spending 0% of the observation time engaged in that same behaviour.

**Table 5:** Odds ratios (OR) for treatment effects on the proportions of time the focal animals spent exhibiting state behaviours during the active and inactive continuous observation periods.

**Behavior**	**Active**						**Inactive**					
	**Main effect of 0.01 increase in** ***k*****-value**			**Main effect of unrestricted trough compared to restricted**			**Main effect of 0.01 increase in** ***k*****-value**			**Main effect of UR trough compared to R**		
	**OR**	**95% CI**	***p-*value**	**OR**	**95% CI**	***p-*value**	**OR**	**95% CI**	***p-*value**	**OR**	**95% CI**	***p-*value**
Standing	0.45	0.29–0.71	0.001	1.72	1.04–2.88	0.036	1.09	0.76–1.57	0.632	0.89	0.52–1.52	0.679
Lying				0.52	0.29–0.92	0.026	0.85	0.57–1.25	0.399	1.05	0.60–1.84	0.855
Grooming	0.64	0.45–0.90	0.011				1.44	0.83–2.49	0.195	1.74	0.72–4.20	0.220
Locomotion	1.06	0.79–1.42	0.704	1.13	0.77–1.66	0.526	1.91	1.31–2.78	0.001	0.75	0.42–1.34	0.339
Nesting	0.77	0.58–1.03	0.083	0.88	0.56–1.38	0.576	0.91	0.54–1.53	0.721	1.90	0.66–5.49	0.238
Interaction Conspecific	0.90	0.63–1.29	0.565	0.98	0.54–1.77	0.942	1.75	1.04–2.94	0.036	0.53	0.22–1.28	0.161
Interaction pen	0.85	0.52–1.40	0.523	1.09	0.62–1.94	0.761	1.32	0.77–2.25	0.309	0.89	0.45–1.78	0.752
Interaction water trough	0.86	0.42–1.77	0.686	1.65	0.68–4.05	0.270	1.30	0.67–2.51	0.443	2.61	1.09–6.23	0.031
Interaction feed trough	0.70	0.38–1.30	0.260	4.90	2.13–11.26	0.001	1.40	0.80–2.44	0.240	2.28	1.05–4.99	0.038

Where no results are presented, a significant interaction term is retained in the final model.

**Figure 6:**
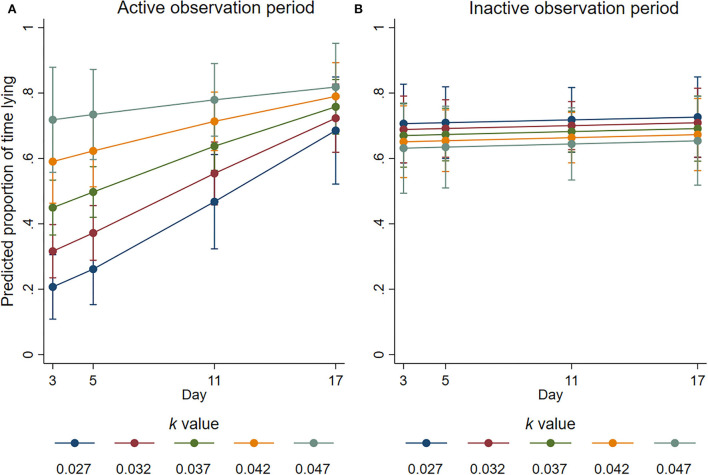
Predicted proportion of time the focal animals housed at different *k*-values spent lying during the **(A)** active and **(B)** inactive observation periods at average values for all the other covariates. Error bars represent 95% confidence intervals of predicted mean numbers.

During the active observation, there was a significant interaction between *k-*value and day (*p* = 0.051) for the proportion of time that the focal animals spent lying (Figure 6). The largest effect was observed on day 3, with the odds of focal animals spending 100% of the observation period lying increasing by a factor of 3.28 for every 0.01 increase in *k*-value (95% CI 1.74–6.19, *p* < 0.001). On days 5 and 11, the odds were increased by a factor of 2.88 (95% CI 1.63 to 5.08, *p* < 0.001) and 1.95 (95% CI 1.16–3.27, *p* = 0.011), respectively, for every 0.01 increase in *k*-value. There was no significant effect of *k*-value observed for the proportion of time spent lying during the active observation on day 17 (estimated factor change in odds = 1.32; 95% CI 0.66–2.65; *p* = 0.432). Activity reduced in all groups as the experiment progressed so that on day 17, the proportion of time spent lying was similar during the inactive and active periods (Figure 6).

There was a significant interaction between trough space allowance and day on the proportion of time spent grooming during the active observation period (*p* = 0.031). The odds of all the 3 focal animals with restricted trough space spending 100% of the observation time grooming was estimated as increasing by a factor of 1.09 for each additional day (95% CI 1.02–1.16, *p* = 0.009); with unrestricted trough space, the estimated factor was 0.99 relative to the animals with restricted trough space (95% CI 0.93–1.05, *p* = 0.701). Time spent grooming was decreased with *k-*value; the odds of all the 3 focal animals spending 100% of the observation time grooming was estimated as decreasing by a factor of 0.64 for every 0.01 increase in *k-*value (95% CI 0.45–0.9, *p* = 0.011).

During the active observation, a significant main effect of day was observed for the time focal animals spent interacting with their pen environment. The odds of all three focal animals spending 100% of the observation time interacting with their pen was decreased by a factor 0.91 for each additional day (95% CI 0.87–0.96, *p* = 0.001). Time spent interacting with feed troughs and water troughs was reduced by a factor of 0.88 (95% CI 0.81–0.96, *p* = 0.003) and 0.91 (95% CI 0.86–0.98, *p* = 0.008), respectively, for every additional day. There was no significant effect of day observed for these behaviours during the inactive observation (all *p* > 0.05).

The number of transitions between standing behaviours and lying was largely unaffected by *k*-value during the active (estimated ration of mean counts = 1; 95% CI 0.72–1.38; *p* = 0.976) and inactive (estimated ration of mean counts = 1.32; 95% CI 0.91–1.93; *p* = 0.139) observation periods. Trough space had no meaningful effect on the number of transitions between standing and lying during the active observation (estimated ration of mean counts = 0.89; 95% CI 0.57–1.39; *p* = 0.617), but during the inactive observation, the animals with unrestricted troughs performed 1.86 times the number of transitions between standing behaviours and lying compared to the animals with restricted troughs (95% CI 1.1–3.12, *p* = 0.02).

For the number of transitions between all state behaviours in the active observation period, a significant interaction between *k*-value and day was evident (*p* = 0.06) such that animals with less space transitioned between behaviours more frequently on days 3 and 5. For every 0.01 increase in *k*-value (i.e., more space), the number of behavioural transitions was estimated as decreasing by a factor of 0.63 on day 3 (95% CI 0.43–0.92, *p* = 0.017), and 0.68 on day 5 (95% CI 0.494–0.934, *p* = 0.017). However, the estimated factor on day 11 was 0.86 (95% CI 0.671–1.11, *p* =0 .242) and on day 17 was 1.09 (95% CI 0.727–1.64, *p* = 0.67). A significant effect of *k*-value was observed during the inactive observation period, in which the number of transitions was estimated as increasing by a factor of 1.38 for every 0.01 increase in *k*-value (95% CI 1.01–1.87, *p* = 0.042). There was no significant effect of trough space allowance observed during the active (estimated ratio of mean counts = 1.23; 95% CI 0.86–1.75; *p* = 0.266) and inactive (estimated ratio of mean counts = 1; 95% CI 0.65–1.54; *p* = 0.999) observation periods.

### Live weight

The mean day 18 live weights for animals in each combination of *k-*value and trough space allowance are shown in Table 6. There was evidence of a small effect of stocking density on live weight at day 18, with an estimated increase of 0.28 kg (95% CI 0–0.57) for every 0.01 increase in *k-*value (i.e., more space) (*p* = 0.053). There was no significant effect of trough space (difference between means = 0.26 kg; 95% CI −0.14 to 0.66 kg; *p* = 0.208).

**Table 6:** Day 18 live weights (in kg) in each combination of stocking density and trough space allowance (mean ± SD; *n* = 72 sheep per combination).

**Stocking density (*k-*value)**	**Trough space allowance**		
	**Unrestricted**	**Restricted**	**Pooled trough treatments**
0.027	44.10 ± 6.76	44.19 ± 5.56	44.14 ± 6.17
0.032	44.84 ± 6.11	44.72 ± 7.28	44.78 ± 6.70
0.037	44.81 ± 6.29	44.34 ± 6.60	44.58 ± 6.43
0.042	45.31 ± 6.21	44.98 ± 6.47	45.14 ± 6.32
0.047	45.25 ± 6.17	44.43 ± 6.88	44.84 ± 6.53
Pooled *k*-values	44.86 ± 6.30	44.53 ± 6.55	

### Faecal glucocorticoid metabolites

Baseline FGCM concentration was a strong predictor of subsequent concentrations (*p* < 0.001). There was no significant effect of *k*-value (estimated change in mean for every 0.01 increase in *k*-value = −0.08 ng/g DM, 95% CI −0.32 to 0.15 ng/g DM, *p* = 0.492) or trough space (estimated change in mean for unrestricted compared to restricted troughs = −0.23 ng/g DM, 95% CI −0.56 to 0.1 ng/g DM, *p* = 0.174) on faecal glucocorticoid concentrations on days 6, 12, and 18 collectively (Table 7).

**Table 7:** Baseline FGCM concentrations (in ng/g DM) and the means ± standard deviations of each sheep's change (Δ) on each sampling day relative to its baseline (in ng/g DM).

	***k*-value**	**Baseline**	**Δ Day 6**	**Δ Day 12**	**Δ Day 18**
Restricted	0.027	2.0 ± 0.6	−1.4 ± 0.9	−1.2 ± 1.3	−1.4 ± 0.7
	0.032	3.6 ± 3.0	−0.1 ± 2.8	+0.5 ± 3.2	−0.3 ± 2.7
	0.037	2.3 ± 1.2	−1.7 ± 3.0	−0.7 ± 1.5	−2.0 ± 1.5
	0.042	2.6 ± 1.4	−1.7 ± 2.1	−0.5 ± 1.4	−1.2 ± 1.6
	0.047	2.9 ± 2.5	−0.4 ± 1.3	−0.1 ± 2.1	−0.3 ± 2.3
Unrestricted	0.027	2.4 ± 1.2	−0.9 ± 0.6	−0.8 ± 0.8	−1.4 ± 1.3
	0.032	2.7 ± 2.1	−0.3 ± 1.8	−0.1 ± 1.4	−0.3 ± 1.9
	0.037	2.1 ± 0.8	−1.6 ± 1.4	−1.1 ± 1.9	−1.6 ± 1.1
	0.042	2.6 ± 1.5	−0.9 ± 1.0	−0.8 ± 1.7	−0.5 ± 1.8
	0.047	2.7 ± 1.7	−0.8 ± 2.0	−0.9 ± 2.2	−0.4 ± 1.4
Pooled trough	0.027	2.2 ± 0.9	−1.1 ± 0.8	−0.9 ± 1.1	−1.4 ± 1.0
treatments	0.032	3.2 ± 2.6	−0.2 ± 2.4	+0.2 ± 2.4	−0.3 ± 2.3
	0.037	2.2 ± 1.0	−1.7 ± 2.2	−0.9 ± 1.7	−1.8 ± 1.3
	0.042	2.6 ± 1.4	−1.3 ± 1.7	−0.7 ± 1.5	−0.8 ± 1.7
	0.047	2.8 ± 2.1	−0.6 ± 1.6	−0.5 ± 2.2	−0.4 ± 1.9
Pooled *k*	Restricted	2.7 ± 2.0	−1.0 ± 2.2	−0.4 ± 2.1	−1.0 ± 1.9
values	Unrestricted	2.5 ± 1.5	−0.9 ± 1.5	−0.7 ± 1.6	−0.3 ± 1.6

Results for each combination of k-value and trough space allowance (n = 10 sheep per combination), each k-value (n = 24 sheep per k-value treatment), and each trough space treatment (n = 60 sheep per treatment) are shown.

### Leucocyte counts

The day 18 concentrations of whole blood white cell counts and reference intervals for the sheep are shown in Table 8.

**Table 8:** Day 18 leucocyte, lymphocyte, and neutrophil counts (× 10^6^ cells/ml) and day 18 neutrophil to lymphocyte ratios (NL ratio) expressed as means and standard deviations.

***k*-value**	**0.027**	**0.032**	**0.037**	**0.042**	**0.047**	**Reference interval**
**Variable**	**Restricted trough space**					
Leucocyte	7.0 ± 1.4	5.8 ± 1.5	5.7 ± 1.6	6.6 ± 1.4	5.8 ± 1.9	5.0–14.0^a^
Lymphocyte	4.5 ± 1.0	3.3 ± 1.3	3.4 ± 1.2	3.7 ± 1.0	2.9 ± 0.8	2.0–5.7^a^
Neutrophil	2.2 ± 1.0	2.1 ± 0.8	1.8 ± 0.7	2.5 ± 0.7	2.3 ± 1.1	1.5–8.6^a^
NL Ratio	0.5 ± 0.3	0.7 ± 0.3	0.6 ± 0.2	0.7 ± 0.4	0.8 ± 0.3	0.1–1.2^b^
	**Unrestricted trough space**					
Leucocyte	6.3 ± 1.5	5.7 ± 1.6	6.4 ± 1.2	5.7 ± 1.8	5.5 ± 0.9	5.0–14.0^a^
Lymphocyte	3.5 ± 1.5	3.3 ± 1.1	3.9 ± 1.1	3.5 ± 1.5	3.4 ± 0.8	2.0–5.7^a^
Neutrophil	2.3 ± 0.7	2.0 ± 0.7	2.2 ± 1.0	1.9 ± 0.5	1.9 ± 0.9	1.5–8.6^a^
NL Ratio	0.8 ± 0.6	0.7 ± 0.3	0.7 ± 0.5	0.6 ± 0.2	0.6 ± 0.4	0.1–1.2^b^
	**Pooled trough treatments**					
Leucocyte	6.6 ± 1.5	5.7 ± 1.5	6.0 ± 1.5	6.2 ± 1.6	5.6 ± 1.5	5.0–14.0^a^
Lymphocyte	4.0 ± 1.3	3.3 ± 1.2	3.7 ± 1.2	3.6 ± 1.3	3.2 ± 0.8	2.0–5.7^a^
Neutrophil	2.3 ± 0.8	2.1 ± 0.7	2.0 ± 0.9	2.1 ± 0.6	2.1 ± 1.0	1.5–8.6^a^
NL Ratio	0.7 ± 0.5	0.7 ± 0.3	0.6 ± 0.4	0.7 ± 0.3	0.7 ± 0.3	0.1–1.2^b^
	**Pooled** ***k*****-value treatments**			
	**Restricted trough space**		**Unrestricted trough space**	
Leucocyte	6.15 ± 1.6		5.9 ± 1.4		5.0–14.0^a^	
Lymphocyte	3.6 ± 1.2		3.5 ± 1.2		2.0–5.7^a^	
Neutrophil	2.2 ± 0.9		2.1 ± 0.8		1.5–8.6^a^	
NL Ratio	0.7 ± 0.3		0.7 ± 0.4		0.1–1.2^b^	

Results for each combination of k-value and trough space allowance (n = 10 sheep per combination), each k-value (n = 24 sheep per k-value treatment), and each trough space treatment (n = 60 sheep per treatment) are shown.

^*a*^Reference ranges reported from UC Davis Veterinary Medical Teaching Hospital (30).

^*b*^Reference range reported from Lepherd, Canfield (31).

There was no significant effect of *k-*value or trough space on day 18 leucocyte counts (*p* = 0.2); any effect of stocking density on leucocyte counts was small, with an estimated decrease of 0.22 × 10^6^ cells/ml (95% CI −0.55 to 0.11 × 10^6^ cells/ml) for every 0.01 increase in *k-*value (i.e., more space). The regression results also revealed that any effect of trough space restriction was small (estimated decrease of 0.33 × 10^6^ cells/ml for animals with unrestricted trough space; 95% CI −0.79 to 0.14 × 10^6^ cells/ml; *p* = 0.173).

### Lymphocyte and neutrophil counts

**Figure 7:**
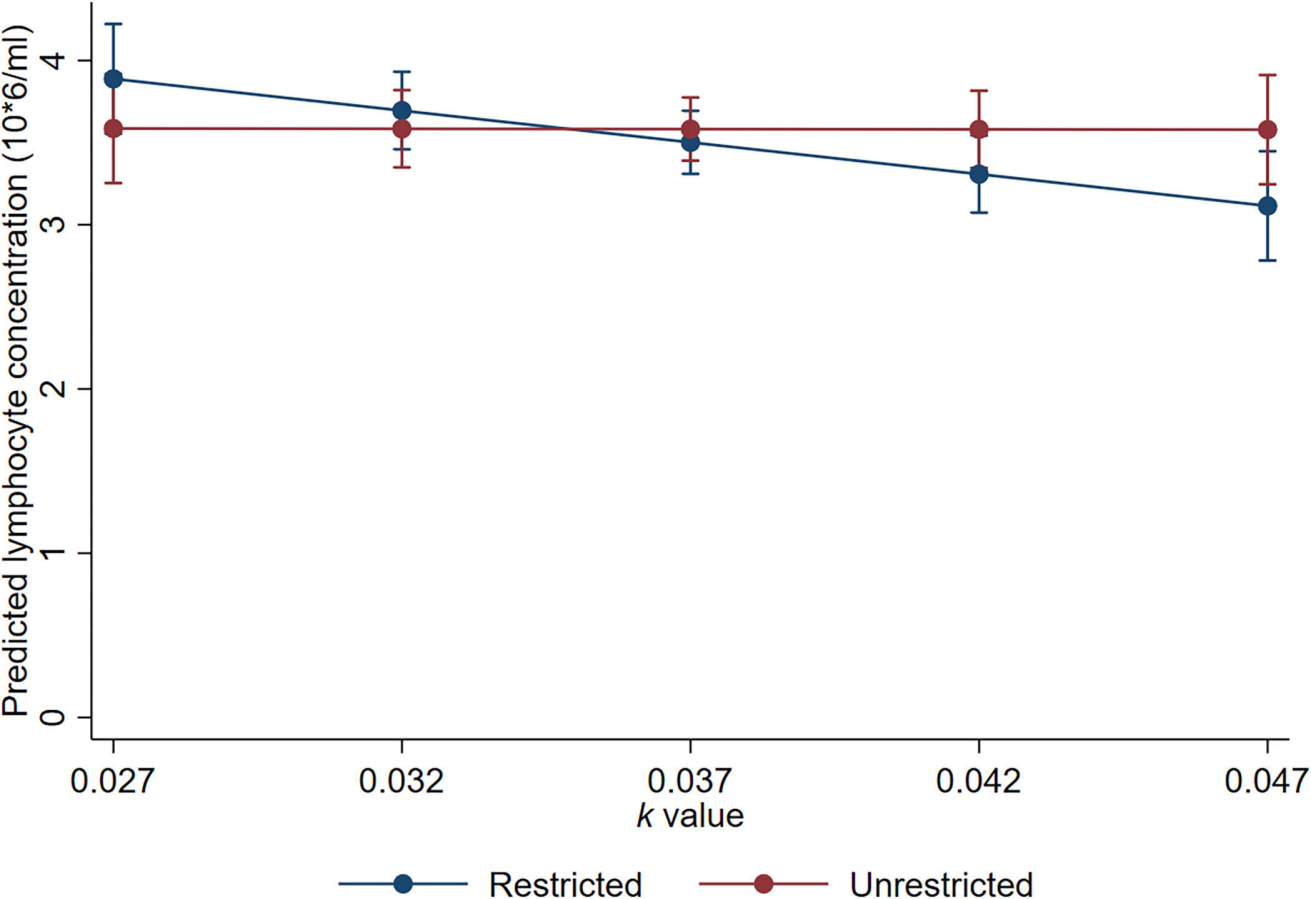
Linearly predicted lymphocyte concentrations for the interaction between trough allowance and *k*-value. Error bars represent 95% confidence intervals of predicted means.

There was evidence of an interaction between *k-*value and trough space allowance for day 18 lymphocyte concentrations (*p* = 0.051). The estimated effect of a 0.01 *k-*value increase was a small decrease of 0.38 × 10^6^ cells/ml for the animals with restricted trough space relative to the animals with unrestricted trough space (95% CI −0.66 to −0.11 × 10^6^ cells/ml, *p* = 0.005), but there was no significant effect of *k-*value for the animals with unrestricted trough space (estimated effect = −0.003; 95% CI −0.27 to 0.27 × 10^6^ cells/ml; *p* = 0.979; Figure 7).

There were no significant effects of *k*-value (estimated effect = −0.15; 95% CI −0.21 to 0.18; *p* = 0.878) or trough space allowance (estimated effect = −0.2; 95% CI −0.47 to 0.07; *p* = 0.153) on neutrophil concentrations at average values for all the other covariates. Furthermore, there were no significant effects of *k*-value (estimated effect = 6% increase, 95% CI −5 to 18%, *p* =0 .332) or trough space allowance (estimated effect = 10% decrease, 95% CI 13–5%, *p* = 0.171) on neutrophil to lymphocyte ratio.

## Discussion

This study was conducted to assess the welfare implications for sheep of five different *k*–value stocking densities of relevance to the Australian live export industry, as well as the welfare implications of restricted trough space. A range of factors that may impact sheep welfare during live export voyages were not present in this study; we have purposefully limited the factors to pen and trough space allowances under thermoneutral conditions to gain a comprehensive understanding of the role of these environmental components in isolation, and future studies should aim to increase environmental complexity and provide information on the impact of other factors such as human activity, wave motion, and warm climatic conditions. The 18-day experimental period is of comparable length to live export voyages that transport sheep from Fremantle, Western Australia to ports of the Middle East, and our results showed that neither reduced pen nor trough space impaired the welfare of sheep for given time period. This was demonstrated by the lack of important effects of *k*-value stocking density or trough space allowance on sheep HPA axis activity, final live weights, or immune cell counts. Additionally, trough space restriction had no important effects on sheep behaviour. However, some effects of *k*-value stocking density on sheep behaviour were observed; important behavioural differences were predominantly observed in the scan sampling analysis of group behaviour and during the active focal observation period. Limited important effects were observed during the inactive observation period because the animals settled into a period of daytime rest and rumination. We observed a higher number of agonistic and displacement interactions for sheep with less space at the start of the trial, as well as a reduced proportion of time spent lying during one observation period, suggesting that the animals took longer to adapt to their environment. Additionally, some preferred lying positions were prevented when stocking density was high (i.e., low *k*-values); when provided with more floor space, a higher number of sheep adopted sternally recumbent lying positions with any number of outstretched legs, and more sheep chose to lie in physical isolation from conspecifics. Importantly, *k*-value had no important effect on the overall ability of sheep to lie; the number of animals lying within a 24-h period and the synchronicity of lying were consistent across all *k*-value treatments. Behavioural changes are the first and most biologically economical response to stressors in most cases (32). Often, such as in the current study, these changes alone are sufficient to manage a stressor (32). The lack of impact on further biological responses suggests behavioural changes were sufficient to allow sheep to cope with their environments, and that impacts on the expression of lying behaviours did not have negative consequences on sheep welfare. It was found that sheep welfare was consistent across all the treatment groups for *k*-value stocking density and trough space allowance but suggests that the provision of additional space facilitates the expression of preferred lying positions and may enable animals to adapt to their environment more quickly.

A free opportunity to lie down is considered a basic requirement of ruminants, and lying is a validated metric of animal behaviour with regard to welfare (33). Reduced space allowance contributed to reductions in the proportion of time the focal animals spent lying during the active period on days 3, 5, and 11, but the effect size was reduced as day progressed, which may reflect adaption to the pen environment. There was no important effect of space allowance on the proportion of time the sheep spent lying during the non-active period. During the active observation, the sheep with less space spent more time grooming themselves compared to those housed at lower stocking densities. For the sheep housed at higher stocking densities in the current experiment, the small reductions in lying time and increases in time spent grooming during the active observation period may reflect the performance of grooming as a redirected behaviour to facilitate sheep coping with their environment in which their ability to lie was inhibited. In a similar manner, with regards to trough space restriction, the proportion of time spent grooming remained consistent for unrestricted trough pens but increased for restricted pens during the inactive observation over the course of the trial. This may reflect the expression of grooming as a redirected behaviour in response to having restricted access to feed. The relationship between self-grooming behaviours and emotional states of animals is unclear (34), but high-levels of self-grooming have been identified as a potential redirected behaviour in cattle (35), and research has found that compared to control animals, cows that had their lying time greatly restricted displayed an increase in the duration and frequency of grooming (36). Importantly, the reduction in lying time for focal animals in high stocking density pens during the active period did not influence the overall number of animals lying within 24 h. Together with the lack of meaningful effect of stocking density on focal animal lying time during the non-active period, this suggests that sheep were able to adjust the times when they could lie which may have prevented lying deprivation from becoming a substantial stressor. Furthermore, we show that stocking density, under the experimental conditions, had no important effect on the synchronicity of lying, which supports previous findings that *k*-values as low as 0.027 facilitate synchronous lying in sheep (5).

Despite stocking density having no important effect on the overall ability of sheep to lie, it did affect the body and head positions expressed by lying sheep, which may have implications for quality of rest, an important consideration with regards to animal welfare (37). In the current study, the proportion of animals lying with outstretched legs was increased by a factor of up to 1.29 for every 0.01 increase in *k*-value, indicating that sheep stretch out their legs when provided with more space. It is therefore evident that sheep may prefer lying positions that take up more space than what a *k*-value of 0.027 permits them to when the group is completely synchronous in lying. It has been suggested that a *k*-value of 0.047 permits animals to adopt a broader range of lying positions (4), and the linear effect of increasing *k*-value resulting in more sheep lying with outstretched legs observed in the current study suggests that this is true and may apply to even greater *k*-values than those tested in the current study. Furthermore, we showed that the number of sheep lying in body contact with conspecifics was reduced when the sheep were provided with more space; every 0.01 increase in *k*-value resulted in 0.71 fewer sheep (of the group of 18) resting in physical contact with a conspecific. In particular, fewer sheep rested their head on a conspecific when more space was provided. These results indicate a preference for some animals to lie in physical isolation when available lying space permits them to do so, which is in agreement with previous research (38, 39). We expected that the sheep provided with more space would lie with their head down more because the sheep had more space to place their head, but in fact, the proportion of sheep lying with their head down was reduced by a factor of 0.82 for every 0.01 increase in *k*-value. Whilst the effect of lying with head down has shown to positively impact brain activity and sleep cycles in sheep (40), the implications of other lying positions, including preferences for leg positions and where animals place their head down, on sleep quality and, subsequently, welfare remain unknown. Our results showing an effect of stocking density on lying position requires further investigation to truly understand the implications on sheep welfare. We identified relationships of inhibited lying positions under thermoneutral conditions, but it is important to consider that animals exposed to warmer conditions during transportation may be further impacted because of the role of body surface area in heat dissipation and exchange between individuals in close contact. In these instances, sheep required to lie in physical contact with one another (due to space restrictions) may experience exacerbated heat stress, which is a significant welfare concern (41).

For confined animals, freedom of movement is determined by space allowance (42). The sheep provided with more space spent a larger proportion of time performing locomotion during the inactive observation period but not during the active observation period. Also, during the inactive observation, increased space allowance resulted in increased number of transitions between all state behaviours. Previous researchers have observed similar results in ewes and suggested that reductions in time spent moving may be due to the physical closeness of their conspecifics and difficulty associated with movement (43). The space required for livestock to transition between standing and lying has been suggested as *k* = 0.047 (4), so the sheep housed with more space (i.e., higher *k-*values) in the current study likely expressed increases in locomotion during the afternoon as a result of being able to perform comfortable behavioural transitions and move more freely around their pen.

We provide evidence that sheep with more space were more likely to engage in positive social interactions and fewer negative social interactions. The sheep housed at higher *k*-values in the current study spent more time engaging in non-agonistic social interactions during the inactive period but not during the active period. Engaging in non-agonistic social interactions has been linked to positive affective states (4), and these results support previous space allowance research (44). Conversely, agonistic interactions and displacement behaviours are considered to indicate negative well-being (3) and competition for resources (45). The animals housed with less space performed a higher number of agonistic (Figure 4) and displacement (Figure 5) interactions during the active observation period on days 3 and 5 but not on days 11 or 17. Whilst an increase in these behaviours may be expected for any group of social animals within the first days of remixing (46), research has shown that the number of such behaviours decreases over time as animals adapt and establish a stable hierarchy (47). From the current results, it is apparent that animals with less space took longer to adapt likely because they experienced additional competition for pen space and associated short-term stress. The proportion of time spent lying during the active observation period was also reduced for the sheep housed at low *k*-values at the beginning of the trial (Figure 6), but this proportion was increased over time so that *k*-value had no effect on day 17, providing further evidence that sheep with less space took longer to adapt. Of note, important effects on agonistic and displacement interactions were not observed when trough space was restricted, suggesting that space allowance is more critical in this context. The reduction in negative social interactions and increased positive social interactions observed when sheep were provided with more space suggests that greater space allowance may have reduced stress and permitted opportunities for positive experiences and affective states (4).

Limited effects of trough space allowance on sheep behaviour were observed in the current study; however, during the active and inactive observation periods, the animals provided with more trough space spent a significantly larger proportion of time interacting with their feed trough. This contradicts the expectation that sheep with restricted troughs would spend more time interacting with their feed trough as a result of potentially not being able to access feed prior to the observation and continuing to seek feed throughout the day to satisfy a behavioural need. Feed troughs were 1.8 m longer in unrestricted pens compared to restricted pens, and this difference likely contributed to this unexpected result. Future studies should investigate the behaviour of individual animals during competitive feeding periods, rather than the effects at the group level outside of feed times that were investigated here, to further clarify the effects of restricted feed trough space.

The lack of important effects of *k*-value stocking density or trough space allowance on indicators of biological functioning, FCGM concentrations, live weights, and immune cell counts, suggest that despite the behavioural effects observed, the sheep did not perceive pen or trough space restriction to be an ongoing stressor in the experimental context. There was some evidence that longer exposure to the environmental conditions may have greater impact on welfare such that reduced pen space allowance was associated with slight reduction in day 18 live weight. This suggests that the sheep housed at higher stocking densities may have experienced short-term stress contributing to minor increases in energy mobilisation. Such changes in energy mobilisation in response to stress may occur through the actions of glucocorticoids involved in the hypothalamic-pituitary-adrenal (HPA) axis response or through behavioural responses to stress. However, we did not observe an effect of stocking density on FGCM concentrations at any time point. Low concentrations of FGCM were observed across the entire cohort of focal animals compared to other studies that have investigated the relationship between FGCM concentrations and pain (48, 49) or 24-h road transport (50). This suggests that the marginal impact on day 18 live weights may have been reflective of the increased competition and aggression seen in the sheep housed with less space at the start of the trial rather than increased HPA axis activity. Of note, the lack of relationship between day 18 live weights and trough space allowance suggests that aggression and competition may be related to other factors that are not related to food (i.e., preferred lying posture and/or establishing a social hierarchy). We unexpectedly observed that increased pen space allowance resulted in a very small decrease in lymphocyte concentrations for restricted feed trough pens but had no important effect on unrestricted pens. This contradicts the expectation that a lower-stress environment (i.e., unrestricted trough space) will contribute to higher lymphocyte concentrations, but these concentrations nearly entirely fell within the expected reference range (31), so the effect likely has no important consequences on the biological functioning and welfare of sheep.

Appropriate interpretation of these results relies upon the consideration of several important factors, such as the range of *k*-value stocking densities investigated, the duration of the study, and the lack of additional stressors that may contribute to a more complex environment and increase the implications of pen and trough space restrictions in the context of real live export voyages. Of note, the effects of *k*-value on all the outcome variables were found to be linear in nature. The minor increases in behavioural expression achieved by providing more space may extend beyond the experimental *k*-values; conversely, the expression of some behaviours may be further reduced at *k*-values below 0.027. *k*-values lower than 0.027 may also contribute to increased physiological stress response and impairments in biological functioning if behavioural responses are insufficient in enabling animals to cope with their environment. Furthermore, it is important to consider that the treatments were imposed for 18 days; animals may experience additional stress relative to each *k-*value and trough space allowance for intensive housing periods that persist beyond this or when additional stressors are also imposed. Additional factors that may induce stress during this mode of transport such as hotter climates, human activity, and movement associated with ocean swell were not present; it is important to test how stocking density interacts with other stressors and how stressors accumulate to affect sheep welfare in future studies.

## Conclusion

The aim of this study was to assess the welfare implications for sheep of five *k*-value stocking densities with either unrestricted or restricted trough allowance. Trough space restriction did not contribute to stress or impaired welfare in the current study. Stocking density had an effect on some lying positions and agonistic and displacement behaviours, suggesting that the sheep were unable to lie in preferred positions and took longer to adapt to their environment when housed with less space (i.e., at low *k*-values). However, we also provide some evidence that the space restriction was not an ongoing stressor, reflected by the lack of changes in biological functioning. Sheep welfare was not impacted by the experimental *k*-values based on the welfare indicators assessed in this study. However, the provision of additional space was beneficial in reducing the time taken for the animals to adapt to their environment and allowing the expression of preferred lying positions, which may have important implications for sheep welfare not identified in the current study (i.e., implications of quality rest and sleep). This research has provided foundational knowledge of stocking densities for intensive housing and sea transport, but the conclusions must be interpreted in the context of ambient temperature and experimental conditions, and further research is required to determine the cumulative effects of environmental conditions during live export (i.e., heat, humidity and wave motion) and the relationship between space use and sheep welfare.

## Data availability statement

The raw data supporting the conclusions of this article will be made available by the authors, without undue reservation.

## Ethics statement

The animal study was reviewed and approved by the CSIRO Chiswick Animal Ethics Committee under the NSW Animal Research Act, 1985 (approval ARA 20/05). Written informed consent was obtained from the owners for the participation of their animals in this study.

## Author contributions

FC, LT, and PT: research funding acquisition and original draft editing. LT: project administration. LT, FC, PT, and BM: conceptualisation and methodology. BM and JM: data curation and formal analysis. BM: original draft preparation. BM, LT, BD, and FC: sample and data collection. BM, LT, BD, FC, and MA: experimental conduct. BM, JM, PT, LT, and FC: interpretation of data. All authors approved the final version of the manuscript.

## Funding

This study was funded by Meat and Livestock Australia Pty Ltd., North Sydney, NSW, Australia, LiveCorp North Sydney, NSW, Australia, and the Australian Government, Canberra, ACT, Australia (W.LIV.0299).

## Conflict of interest

Author JM was employed by Jemora Pty Ltd. The study received funding from Meat and Livestock Australia Pty Ltd. The funder was not involved in the study design, collection, analysis, interpretation of data, the writing of this article or the decision to submit it for publication. All authors declare no other competing interests.

## Publisher's note

All claims expressed in this article are solely those of the authors and do not necessarily represent those of their affiliated organizations, or those of the publisher, the editors and the reviewers. Any product that may be evaluated in this article, or claim that may be made by its manufacturer, is not guaranteed or endorsed by the publisher.
