# Corrigendum: Stocking density, restricted trough space, and implications for sheep behaviour and biological functioning

**DOI:** 10.3389/fvets.2022.1077412

**Published:** 2022-11-22

**Authors:** Bonnie T. Mayes, L. Amy Tait, Frances C. Cowley, John M. Morton, Brendan P. Doyle, Muhammad A. Arslan, Peta S. Taylor

**Affiliations:** ^1^School of Environmental and Rural Science, University of New England, Armidale, NSW, Australia; ^2^Jemora Pty Ltd., East Geelong, VIC, Australia

**Keywords:** live export, faecal glucocorticoid metabolites, lying positions, allometry, ruminant welfare

In the published article, there was an error in Figures 2, 3 as published. The y-axes values for these figures had been calculated incorrectly. The corrected Figures 2, 3 and their captions appear below.

**Figure 2:**
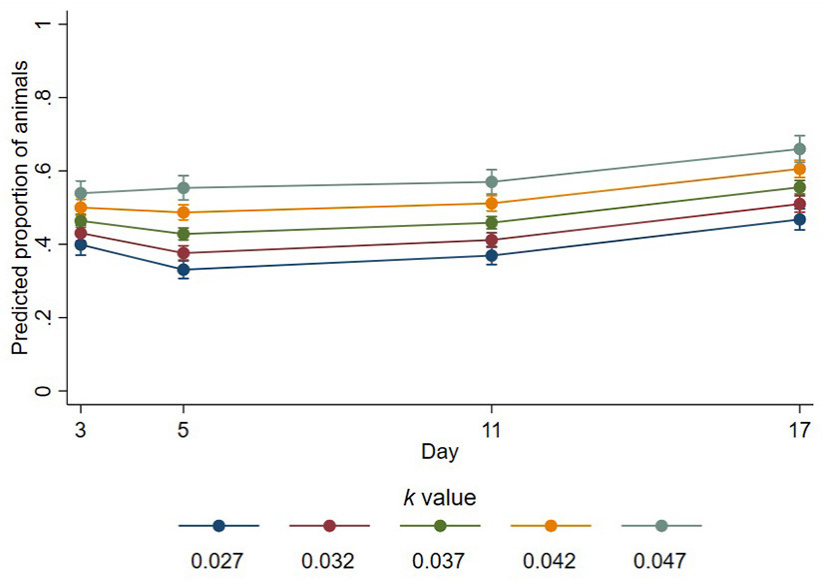
Predicted proportions of lying animals that had outstretched legs at different *k*-values across days. Error bars represent 95% confidence intervals of predicted proportions. Predicted proportions were calculated as predicted numbers divided by the average number of sheep lying at each time point (12.48).

**Figure 3:**
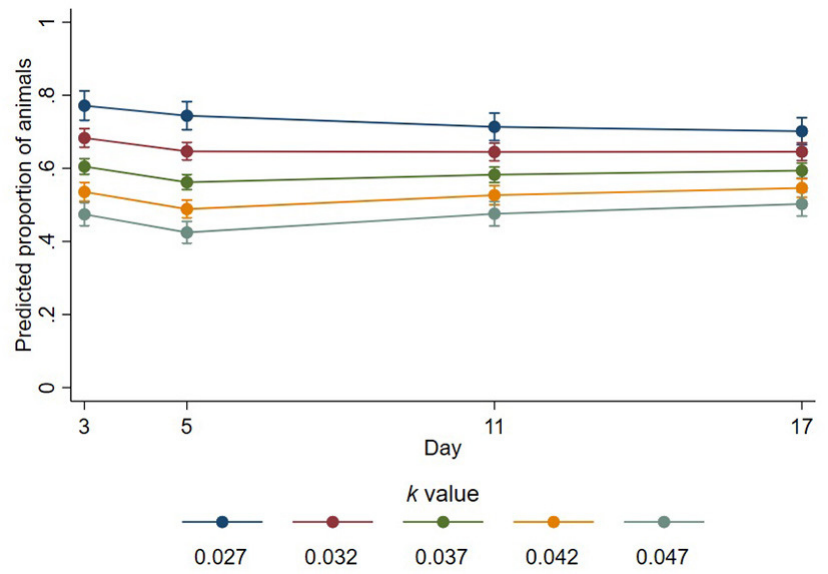
Predicted proportions of animals lying with their head down that place their head on a conspecific at different *k*-values across days. Error bars represent 95% confidence intervals of predicted proportions. Predicted proportions were calculates as predicted numbers divided by the average number of sheep lying at each time point (6.69).

In the published article, there was an error in the **Conflict of interest statement**. The original statement was:

“Author JM was employed by Jemora Pty Ltd. The study received funding from Meat and Livestock Australia Pty Ltd. The remaining authors declare that the research was conducted in the absence of any commercial or financial relationships that could be construed as a potential conflict of interest.”

The corrected statement is:

“Author JM was employed by Jemora Pty Ltd. The study received funding from Meat and Livestock Australia Pty Ltd. The funder was not involved in the study design, collection, analysis, interpretation of data, the writing of this article or the decision to submit it for publication. All authors declare no other competing interests.”

The authors apologize for this error and state that this does not change the scientific conclusions of the article in any way. The original article has been updated.

## Publisher's note

All claims expressed in this article are solely those of the authors and do not necessarily represent those of their affiliated organizations, or those of the publisher, the editors and the reviewers. Any product that may be evaluated in this article, or claim that may be made by its manufacturer, is not guaranteed or endorsed by the publisher.

