# Supplementary material for: Stocking density, restricted trough space, and implications for sheep behaviour and biological functioning
Source: Front Vet Sci. 2022 Sep 28;9:965635. doi: 10.3389/fvets.2022.965635 (PMC9556270; doi:10.3389/fvets.2022.965635)
Supplement: Supplementary file 1 [file Data_Sheet_1.docx]

Supplementary Material

# Supplementary Figures and Tables

**Supplementary Table 1** Mean daily dry-bulb temperature (T_DB_), wet-bulb temperature (T_WB_) and relative humidity (RH) over the 18 day experimental period for runs 1 and 2.

|  | **Run 1** | | | **Run 2** | | |
| --- | --- | --- | --- | --- | --- | --- |
| **Day** | T_DB_ (°C) | T_WB_ (°C) | RH (%) | T_DB_ (°C) | T_WB_ (°C) | RH (%) |
| 0 | 18.9 | 14.7 | 67.9 | 14.4 | 9.8 | 61.8 |
| 1 | 17.4 | 10.6 | 47.2 | 16.2 | 11.2 | 60.6 |
| 2 | 15.6 | 9.2 | 49.2 | 12.8 | 9.9 | 73.2 |
| 3 | 16.2 | 10.9 | 58.2 | 13.3 | 9.6 | 68.8 |
| 4 | 14.1 | 11.3 | 75.4 | 16.7 | 12.1 | 66.9 |
| 5 | 16.3 | 12.4 | 68.0 | 19.0 | 13.9 | 63.1 |
| 6 | 13.9 | 9.3 | 60.2 | 19.2 | 14.1 | 62.0 |
| 7 | 17.1 | 11.6 | 58.5 | 18.1 | 14.1 | 68.1 |
| 8 | 17.4 | 12.2 | 59.4 | 20.7 | 14.7 | 57.5 |
| 9 | 19.0 | 13.0 | 56.5 | 21.4 | 13.8 | 49.9 |
| 10 | 16.9 | 12.8 | 65.7 | 24.4 | 16.7 | 53.0 |
| 11 | 14.6 | 12.5 | 80.4 | 22.7 | 16.6 | 61.2 |
| 12 | 14.1 | 11.7 | 79.0 | 16.8 | 13.0 | 70.6 |
| 13 | 16.9 | 13.3 | 73.1 | 20.3 | 15.2 | 65.7 |
| 14 | 16.3 | 14.1 | 80.6 | 22.5 | 16.1 | 57.7 |
| 15 | 19.4 | 15.4 | 70.4 | 22.5 | 16.3 | 57.5 |
| 16 | 16.9 | 15.1 | 84.3 | 23.9 | 17.3 | 59.6 |
| 17 | 17.6 | 14.7 | 75.9 | 23.2 | 16.3 | 54.2 |
| 18 | 15.9 | 12.6 | 73.5 | 21.0 | 16.0 | 64.0 |

**Supplementary Table 2** Coefficients for Lin’s Concordance correlation and intra-class correlation between the trainer (BM) and observer (MA) for scan sampling behaviours recorded immediately after training and upon conclusion of data collection. Agreement results are based on 56 observation timepoint images.

| **Behaviour** | **Immediately after training** | | **Upon conclusion of data collection** | |
| --- | --- | --- | --- | --- |
|  | **LCC** | **ICC** | **LCC** | **ICC** |
| Standing | 0.999 | 0.999 | 0.995 | 0.995 |
| Lying 1 | 0.902 | 0.888 | 0.673 | 0.827 |
| Lying 2 |  |  |  |  |
| Lying 3 | 0.959 | 0.950 | 0.886 | 0.871 |
| Lying 4 | 0.858 | 0.796 | 0.931 | 0.938 |
| Body 1 | 0.994 | 0.994 | 0.963 | 0.952 |
| Body 2 | 0.933 | 0.907 | 0.617 | 0.638 |
| Head 1 | 0.934 | 0.910 | 0.940 | 0.915 |
| Head 2 | 0.917 | 0.860 | 0.882 | 0.886 |
| Head 3 | 0.888 | 0.880 | 0.806 | 0.745 |

**Supplementary Table 3** Intra-observer reliability of scan sampling behaviours recorded by the observer (MA) for 57 observation timepoint images, in terms of Lins concordance coefficient (LCC) and intraclass correlation (ICC)

| **Behaviour** | **Intra-observer reliability** | |
| --- | --- | --- |
|  | **LCC** | **ICC** |
| Standing | 0.998 | 0.998 |
| Lying 1 | 0.955 | 0.924 |
| Lying 2 |  |  |
| Lying 3 | 0.964 | 0.939 |
| Lying 4 | 0.831 | 0.808 |
| Body 1 | 0.994 | 0.992 |
| Body 2 | 0.872 | 0.796 |
| Head 1 | 0.946 | 0.922 |
| Head 2 | 0.901 | 0.885 |
| Head 3 | 0.883 | 0.859 |
